# Knowledge, Attitude, and Practice on Antibiotic Use and Resistance Among Undergraduates, Pokhara Metropolitan, Nepal

**DOI:** 10.1155/bmri/9928264

**Published:** 2025-02-10

**Authors:** Grinsun Sharma, Shishir Paudel, Anisha Chalise, Biswash Sapkota, Nirmal Raj Marasine

**Affiliations:** ^1^School of Biomedical Sciences, Kent State University, Kent, Ohio, USA; ^2^Department of Public Health, CiST College, Pokhara University, Kathmandu, Nepal; ^3^Center for Research on Environment, Health, and Population Activities (CREHPA), Kathmandu, Nepal; ^4^Department of Pharmacy and Clinical Pharmacology, Madan Bhandari Academy of Health Sciences, Hetauda, Nepal; ^5^Department of Pharmacy, CiST College, Pokhara University, Kathmandu, Nepal

**Keywords:** antibiotic use, antimicrobial resistance, KAP, Nepal, undergraduates

## Abstract

**Background:** Antimicrobial resistance (AMR) is a global health problem contributing to increasing rates of morbidity and mortality throughout the world. Inadequate knowledge and misconceptions surrounding antibiotics and their overuse can significantly contribute to the growth and spread of AMR. This study aimed to assess knowledge, attitudes, and practices (KAP) regarding antibiotic use and resistance among undergraduates in Pokhara, Nepal, and examine associations of knowledge and attitudes with demographic factors and specific antibiotic use practices.

**Methods:** A cross-sectional survey was conducted from April to September 2023 among 461 undergraduate students in Pokhara Metropolitan City. Frequency distributions were used to describe participants' KAP regarding antibiotics. Chi-square tests were applied to identify factors significantly associated with knowledge and attitudes toward antibiotics, with a significance level of 5%.

**Result:** Among the total participants, 155 (33.6%, 95% CI: 29.6–38.3) had a lower level of knowledge regarding antibiotics, while a moderate and higher level of knowledge was observed among 208 (45.1%, 95% CI: 40.1–49.5) and 98 (21.3%, 95% CI: 17.5–24.7) students, respectively. Nearly half (53.6%, 95% CI: 48.9–58.2; 50.1%, 95% CI: 45.8–54.4) of the students illustrated positive attitudes and good practices. Factors such as gender, academic discipline, and previous education on antibiotics were associated with students' knowledge regarding antibiotics, at 5% level of significance (*p* < 0.05). Similarly, a statistically significant relationship existed between students' academic discipline, past courses, and their attitudes toward antibiotics. The knowledge of the participants on the antibiotic use was found to have a statistical relationship with practice-related attributes such as finishing the antibiotic course even after feeling well, giving less preference to antibiotic for cold (*p* < 0.05). The attitude of the participants toward antibiotics was found to have a statistical relation with practices such as consulting a doctor for antibiotics and finishing the course of antibiotic (*p* < 0.05).

**Conclusion:**A large proportion of undergraduates have moderate to high knowledge regarding antibiotic use, while only half had a positive attitude and good practice toward antibiotic use, suggesting a large gap existing in awareness of antibiotic resistance and rational use.

## 1. Introduction

Antimicrobial resistance (AMR) presents a significant global health challenge that results in ineffective treatments, extended hospital stays, increased healthcare expenses, and high rates of morbidity and mortality throughout the world [1, 2]. Microbial resistance could develop as microorganisms such as bacteria, viruses, fungi, and parasites evolve certain mechanism which reduces the effectiveness of drugs or other substances intended to treat or prevent infections [3]. Among various AMRs, antibiotic resistance is particularly alarming globally, as antibiotics are becoming less effective, and countries are experiencing constant shortages regardless of their national economy [2]. A systematic analysis based on 204 countries and territories on the global burden of bacterial AMR highlighted that globally bacterial AMR was associated with almost 4·95 million deaths in the year 2019 [4].

Inadequate knowledge and misconceptions surrounding antibiotics and their overuse can significantly contribute to the growth and spread of AMR [5]. Furthermore, the inappropriate prescribing of antibiotics can also lead to antibiotic resistance [6]. A recent survey revealed that approximately one-third (33.7%) of the individuals in low- and middle-income countries do not have proper understanding of antibiotics and their mechanism of action [7]. In developing nations, the scarcity of diagnostic tools, easy accessibility of antimicrobials without a prescription, and a deficiency in suitable drug regulatory contribute to the problem of AMR [8].

Nepal has been observing multiple cases of antibiotic resistance such as an increasing rate of urinary tract infections (UTIs) caused by ampicillin, cotrimoxazole, amikacin, and nitrofurantoin-resistant strains of *Escherichia coli* [9], *Pneumococcal* disease resistance to ciprofloxacin and cotrimoxazole [10], and skin infections by *methicillin-resistant Staphylococcus aureus (MRSA)* [11]. In response to the problem of AMR, Nepal drafted the National Action Plan for Antimicrobial Resistance 2021–2026, referencing the Global Action Plan on Antimicrobial Resistance (GAP-AMR) while adopting the “One Health” approach [12]. The Department of Drug Administration (DDA) has taken some strict measures in the registration of new drugs' manufacturing or import [13]. Despite the measures taken by the Government of Nepal to address AMR and promote the rational use of antibiotics, the AMR challenge persists and continues to escalate. This could primarily be attributed to the community's limited understanding of antibiotic use and resistance, as several prior studies in Nepal have identified gaps in knowledge, attitudes, and practices (KAP) related to antibiotics [14–17].

University students, particularly those in undergraduate programs, are at a crucial stage in their educational and professional journeys. They constitute the upcoming workforce and social influencers who can raise awareness within a broader community. Assessing KAP related to antibiotics in this group is essential for promoting rational use of antibiotics [18]. Self-medication practices such as obtaining medicines without prescriptions, sharing medication with others, reusing old prescriptions, and not following prescribed instructions regarding dosage and duration are other major reasons for increasing AMR [19], which is also linked to the KAP related to antibiotics. Self-medication is also a common practice among students due to time constraints, repetitive symptoms, self-diagnostic confidence, and past ineffectiveness of prescribed medications [20]. This makes university study an important population group to be considered for antibiotic use and resistance, but there are limited studies that have focused on antibiotic-related KAP in this population, while most of them are based on medical students [17, 21, 22]. Thus, this study is aimed at assessing the KAP regarding antibiotic use and resistance among undergraduate students in Pokhara Metropolitan, Nepal. Additionally, it examines associations between KAP levels and demographic factors, including age, gender, academic discipline, and prior exposure to antibiotic-related courses. Further, the study explores the relationship between specific antibiotic use practices and participants' knowledge and attitudes.

## 2. Methods

### 2.1. Study Site

**Figure 1:**
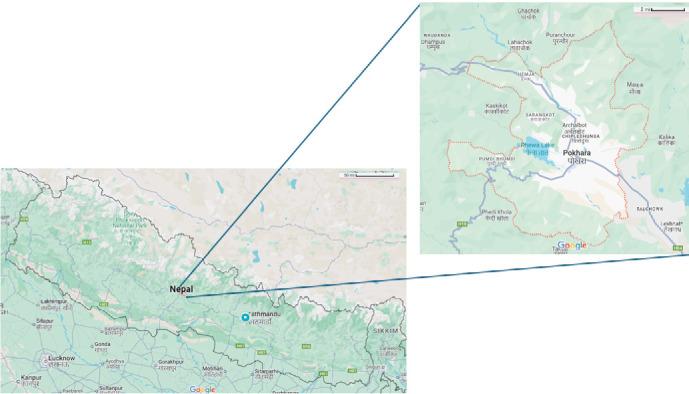
Map of the study area.

This study was executed at Pokhara Metropolitan City, Kaski district, Gandaki Province of Nepal (Figure 1). Pokhara Metropolitan is the largest metropolitan of the country with a population density of 1300/km^2^ and a higher proportion of youth. It is one of the most literate cities with a literacy rate of 81.7% [23].

### 2.2. Study Design

The cross-sectional study was executed between April and September 2023, involving 461 undergraduate students residing inside the Metropolitan City.

### 2.3. Sample Size and Sampling Technique

The undergraduate students residing in the city for at least 6 months prior to the data collection were eligible to participate. The sample size for the study was drawn using the Cochran formula for proportion (*Z*^2^*pq*/*e*^2^). A previous study from Nepal revealed that almost half (50%) of the students had a good attitude toward antibiotic use [17]. Using this past proportion at a 95% confidence interval, the initial sample size was calculated to be 385, which was optimized to 461, adjusting 20% nonresponse rate. To increase the representation from each academic group in the sample, a stratified random sampling technique was followed, for which major undergraduate programs available inside Pokhara Metropolitan were identified. Every accessible program was classified into strata based on their streams such as management, allied sciences, health science, engineering, and others covering humanities, education, and arts. The participants were selected based on the proportion of students enrolled at each stratum covering seven academic disciplines from eight different educational institutions.

### 2.4. Data Collection

The data was collected after acquiring approval from each academic institution and written informed consent from the undergraduates. Data collection involved a self-administered questionnaire, divided into four sections. The initial section of the questionnaire covered sociodemographic characteristics of the participants including age, gender, and current academic majors/discipline. The second section consisted of a 5-point Likert scale with 10 items to assess the participants' knowledge of antibiotic use and resistance. Similarly, the third section consisted of six items on a 5-point Likert scale to assess their attitude toward antibiotic resistance, and the fourth section contained five statements on a 5-point Likert scale to assess their antibiotic use or practice. The data collection tool is attached as supporting information (S1).

### 2.5. Outcome Variable

The students were provided with a few statements and asked to provide their responses on a Likert scale to evaluate the level of KAP on antibiotic use. Participants who selected “strongly agree” or “agree” in the 5-point Likert were considered to have agreed, while those who selected “strongly disagree” or “disagree” were considered to have disagreed and neutral response as neutral to simplify the analysis process. For knowledge, each accurate response was awarded 1 point, while neutral or incorrect responses received 0 points. The maximum achievable score for the knowledge section was 10. Similarly, to assess attitude, six statements were used where acceptance of each positive statement was noted as 1 while neutral or negative responses received 0. The maximum achievable score for attitude was 6. Similarly, to assess practice, five statements were used and participants reported their practice as in 5-point Likert ranging from always to never. Each positive practice was scored 1 and negative practice was scored 0. In all three scales, both positive and negative statements were used, and all the negative statements were reversed coded during analysis before computing the total score of each respective scale. The normality test of all three scales was performed. The cutoffs were set to categorize the score, where for knowledge score of 0–3 was used to denote a low level of knowledge followed by moderate (4–5) and high (6–10); for attitude, score of 0–2 represents negative attitude while score of 3–6 represent positive attitude; and for practice 0–2 represent poor practice while 3–5 represented good practice. To ensure the validity and reliability of the tool to assess KAP, an intensive literature review was performed, the expert pharmacists were consulted, and the research team comprised pharmacy and public health experts. The team also took reference from the questionnaire developed and previously used by Khan et al. [24] and Awad AI et al. [25] and modified it based on Nepalese context. The tool was pretested among 10% of the total sample population (48 students) of Pokhara Metropolitan City who did not fall under the randomly drawn sample and the tool was slightly altered before final administration.

### 2.6. Statistical Analysis

The data obtained was entered into EpiData software Version 3.1 and analyzed using Statistical Package for Social Science Version 20. Frequency distribution for each statement was assessed to understand the extent of KAP regarding antibiotics. The relationship between the independent and dependent variables was examined using a chi-square test, at a 5% level of significance (*p* < 0.05).

## 3. Result

Four hundred sixty-one students participated in the study, providing complete responses to each question. Out of 461 participants, 215 (46.6%) were males and 246 (53.4%) were females. The average age of the students in years was 21.30 ± 1.62, with ages ranging from 17 to 30 years. Almost half (51.6%) of the students reported having studied antibiotics in their past studies (Table 1).

In terms of antibiotic knowledge, more than third of a quarter (84.8%) knew different antibiotics are needed to cure different diseases. Similarly, less than half (42.3%) said antibiotics could kill the normal flora on human skin and the gut. Likewise, almost half of the students (59%) knew that excessive use of antibiotics could increase the bacterial resistance antibiotics, and almost half (56%) took it as a global problem. Furthermore, less than half (46%) of the students believed that humans could develop resistance to antibiotics, and nearly half (50.1%) believed that antibiotics work against viruses (Table 2). It was found that out of 461 students, nearly one-third (33.6%, 95% CI: 29.6–38.3) of the students had a lower level of knowledge, while almost half reported moderate knowledge (45.1%, 95% CI: 40.1–49.5) followed by higher knowledge (21.3%, 95% CI: 17.5–24.7).

In terms of attitudes, approximately 54.7% of the students indicated that they believed it was necessary to finish the entire course of treatment even if they started feeling better. In comparison, only one-fourth (24.9%) stated that skipping a few doses will not contribute to developing antibiotic resistance. More than half (64.6%) of the students considered the everyday use of antibiotics unsafe. In comparison, only 39.3% of the students acknowledged that they might be contributing to antibiotic resistance while using antibiotics (Table 2) Similarly, in the case of attitude toward antibiotic resistance, nearly half (46.4%, 95% CI: 41.8–51.1) of the students reported a negative attitude toward antibiotic resistance while half (49.9%, 95% CI: 45.6–54.2) of the students noted to have poor practice (Table 3).

When it came to practices around antibiotics, a majority (75.1%) of the participants practice consuming antibiotics only after consulting a doctor. In comparison, 19.5% practice consuming antibiotics with or without a prescription, and 5.4% practice using antibiotics without seeking a doctor or rarely seeking a prescription. Furthermore, only half (52.5%) of the students had practiced completing their course of treatment with antibiotics even after they felt better. In comparison, nearly one-fifth (21.5%) of the students had never completed their treatment after feeling better (Table 2). In the case of overall practice, nearly half (50.1%, 95% CI: 45.8–54.4) of the students reported a good practice of antibiotic use.

In regard to the factors associated with KAP regarding antibiotics, the bivariate analysis revealed that gender, academic discipline, and prior exposure to courses related to antibiotics were significantly associated with knowledge regarding antibiotics (*p* < 0.05). Similarly, attitude toward antibiotic use and resistance was found to have a statistically significant relationship with academic discipline and prior exposure to courses related to antibiotics (*p* < 0.05). Likewise, age and prior exposure to courses related to antibiotics were the factors associated with practice (*p* < 0.05). It was observed that younger participants (< 20 years) have better practices of antibiotic use (69.0%) compared to older participants, particularly those over 23 years, with 58.5% displaying poor practices. However, no significant association was observed between age and both knowledge and attitude (Table 4).

It was further examined if participants' level of knowledge and attitude toward antibiotics have any relationship with their pattern of antibiotic use or practice, which revealed some interesting findings. The participants' knowledge of antibiotics use was found to have a statistical relationship with practice attributes such as finishing the course of antibiotics even after feeling well, giving less preference to antibiotics for colds (*p* < 0.05). However, their level of knowledge did not seem to have any relation with their practices such as consulting a doctor for antibiotics, taking proper counseling before use, and checking for expiry dates. Similarly, regarding their attitude and practice, their attitude toward antibiotics was found to have a statistical relation with their practice such as consulting doctors and completing the antibiotic course (*p* < 0.05). However, practices such as taking proper counseling, checking for expiry dates, and preferring antibiotics for colds were not linked with their attitude (Table 5).

## 4. Discussion

The study revealed that most participants demonstrated a moderate level of knowledge regarding antibiotics. They knew different antibiotics were required to treat various ailments and demonstrate effectiveness in combating bacterial infections. Almost half of the participants agreed that improper antibiotic use could contribute to AMR. However, the proportion of the participants who agreed with this statement was relatively lower than that of past studies conducted among medical students [24, 26–28]. This can be due to the gap in knowledge regarding antibiotics among students from different disciplines as past studies were mostly focused on health science while this study covered diverse academic backgrounds; that might be the reason that a significant relationship exists between participants' current academic discipline and their knowledge regarding antibiotics.

The findings indicate that age was not significantly associated with knowledge and attitude regarding antibiotics, although participants under 20 years had a slightly higher proportion of high knowledge than those over 23 years. However, age was significantly associated with practice, with participants under 20 demonstrating better practices. Similar findings have been observed in prior studies, where younger age groups often show better healthcare compliance than older groups, though some studies report that older students, potentially due to greater educational exposure, exhibit better antibiotic attitudes and practices [6, 25, 29, 30]. We can assume that the lack of association between age and knowledge or attitude in this study might have resulted from the limited integration of antibiotic-related content in their curriculum. The better practices observed among students under 20 years of age might be due to the greater reliance of younger students to their family members for health-related decisions, with close family monitoring and guidance influencing their medication use. These results underscore the importance of age-targeted interventions, focusing on foundational education for younger students and promoting responsible self-reliant practices among older students. Further research is needed to address the discrepancies observed in different studies and explore age-specific factors influencing antibiotic KAP for a more comprehensive understanding.

The proportion of the participants with moderate knowledge and positive attitudes toward antibiotics was high in this study; however, the participants exhibited some uncertainty regarding antibiotic use. Almost half of the participants believed that antibiotics are efficacious against viral infections, and more than half agreed that antibiotics speed recovery from coughs and colds. These findings are consistent with various studies where most participants shared similar beliefs and used antibiotics for viral illnesses, coughs, colds, and fevers [25, 31–36]. Studies suggest these beliefs further lead to a misconception of antibiotics as a standard and safe drug, contributing to the inappropriate high use of antibiotics [24, 31].

Globally, self-medication with antibiotics has been a significant driver of antibiotic resistance [37–39]. Self-medication practices among university students have been highlighted by numerous studies throughout the world regardless of their national economy and diverse healthcare system [36, 38, 40–42]. In this study, however, it was observed that the self-medication practices were relatively low at 5.4%. This might be due to several reasons, such as in Nepalese households, people tend to contact their family members or relatives who are medical professionals to consult about medicines, and in some cases, people tend to confuse community pharmacies as doctors due to improper counseling. While antibiotic use practices overall were satisfactory, more than one-third of the participants reported stopping antibiotics without completing the full course. Nearly half of the participants reported unnecessary use of antibiotics could result in antibiotic resistance, while nearly a quarter agreed using antibiotics in farming could lead to AMR in humans and acknowledged that they contribute to AMR each time they use antibiotics. These findings align with the study conducted among secondary school and university students at Braga [31]. In this study, however, about a quarter of the participants shared their belief that skipping a few doses would not cause the development of AMR. Various studies have recorded these misconceptions about antibiotic use, which can lead to a patient's risk of experiencing a relapse with pathogenic bacteria that are resistant to treatment [25, 43]. This issue has become a critical threat to global health [40, 42]. The significant association was also observed between the current academic disciplines, past studies on antibiotics, and the higher level of knowledge and positive attitude toward antibiotic use and resistance. This study also suggests that the level of KAP about antibiotics can be changed by introducing courses on antibiotics and infectious diseases with an emphasis on bacterial and viral pathogens [31].

The students with a higher level of knowledge and positive attitude demonstrated to have better practices of antibiotic use, such as frequently consulting doctors and completing the course of antibiotics than those with inadequate knowledge and negative attitudes, but other practice-related attributes were found not being associated with the knowledge and attitude. Similar findings were shared by a past study from Kuwait where participants' knowledge of antibiotics and their self-medication practices were not significantly associated [25]. Interestingly, it was observed that participants with little knowledge of antibiotics were more inclined to seek advice from a physician. A similar finding was observed in a study conducted in China; participants with little understanding of antibiotics demonstrated a significant inclination to consult a physician, be prescribed, self-medicate, and use antibiotics as a preventive measure [41]. These findings underscore the pivotal role of antibiotic-related knowledge and attitudes in shaping responsible antibiotic use practices among students.

## 5. Strength and Limitation of the Study

Despite being one of the few studies from the regions assessing KAP related to AMR, covering diverse undergraduate students from various disciplines for a better understanding of the issue across academic disciplines, this study is not free from its limitations. Nepal is a diverse nation with various cultures, languages, and literacy rates across the country; our study being conducted in a single region might fail to represent the whole Nepalese undergraduate population. The study was based on a self-reporting approach and we did not examine the actual practice of the participants; thus, there can be some chances of social desirability bias, as students may share what they thought was socially acceptable rather than their actual attitudes and habits regarding antibiotic use.

## 6. Conclusion

This survey found that most participants showed moderate knowledge and a positive attitude toward antibiotics. Still, some participants used antibiotics for inappropriate reasons and in a wrong way. Students with higher knowledge scores have a more positive attitude and better practice of antibiotic use. A higher knowledge score was noted among the students from the health science discipline or those who had previously studied antibiotics. This study highlights the need to introduce awareness interventions, such as initiating introductory courses on antibiotics and infectious diseases at the school level, to improve KAP attributes on antibiotics and prevent their misuse.

## Figures and Tables

**Table 1:** Sociodemographic characteristics of the participants (*n* = 461).

**Variables**	**Frequency**	**Percentage**
*Age*		
< 20 years	42	9.1
20–23 years	378	82.0
> 23 years	41	8.9
*Gender*		
Male	215	46.6
Female	246	53.4
*Academic major*		
Health sciences (nursing and public health)	55	11.9
Engineering	84	18.2
Management	207	44.9
Allied sciences	57	12.4
Others (humanities, law, education)	58	12.6
*Studies about antibiotics in the past*		
Yes	238	51.6
No	223	48.4

**Table 2:** Knowledge regarding antibiotic use and resistance.

**SN**	**Knowledge regarding antibiotics**	**Agree (%)**	**Neutral (%)**	**Disagree (%)**
K1	Various antibiotics are required to treat various ailments	391 (84.8)	50 (10.8)	20 (4.3)
K2	Antibiotics demonstrate effectiveness in combating bacterial infections	343 (74.4)	84 (18.2)	34 (7.4)
K3	Antibiotics can eliminate essential bacteria that typically reside on the skin and within the gastrointestinal tract	195 (42.3)	177 (38.4)	89 (19.3)
K4^a^	Antibiotics help the recovery from all coughs and colds	296 (64.2)	69 (15.0)	96 (20.8)
K5^a^	Antibiotics are effective against all coughs and colds	233 (50.5)	129 (28.0)	99 (21.5)
K6^a^	Antibiotics work against viral infections	231 (50.1)	119 (25.8)	111 (24.1)
K7	Overuse of antibiotics can grow bacterial resistance toward antibiotics	272 (59.0)	79 (17.1)	110 (23.9)
K8	Antibiotic resistance is a global concern	258 (56.0)	147 (31.9)	56 (12.1)
K9	Humans could develop resistance to antibiotics	212 (46.0)	165 (35.0)	84 (18.2)
K10^a^	The use of antibiotics in animals has no effect in humans	106 (23.0)	144 (31.2)	211 (45.8)
	**Attitude toward antibiotics**			
A1^a^	Taking antibiotics during a cold is crucial to prevent the progression of a more severe illness	204 (44.3)	50 (10.8)	207 (44.9)
A2	It is recommended to complete the entire course of antibiotic treatment even if I start to feel better	252 (54.7)	62 (13.4)	147 (31.9)
A3^a^	Antibiotics aid in a faster recovery when experiencing a fever	246 (53.4)	107 (23.2)	108 (23.4)
A4	Every time I use an antibiotic, I contribute to the emergence of antibiotic resistance	181 (39.3)	188 (40.8)	92 (20.0)
A5^a^	The omission of one or two doses does not lead to the emergence of antibiotic resistance	115 (24.9)	126 (27.3)	220 (47.7)
A6^a^	Antibiotics are widely utilized because they are considered safe medications	90 (19.5)	73 (15.8)	298 (64.6)

	**Practice of antibiotic use**	**Always/usually (%)**	**Sometimes (%)**	**Seldom/never (%)**
P1	I consult a physician/pharmacist before starting an antibiotic	346 (75.1)	90 (19.5)	25 (5.4)
P2	Take proper counseling from pharmacists on how to use antibiotics before buying the medicine	327 (70.9)	82 (17.8)	52 (11.3)
P3	I consistently finish the entire course of antibiotics, even if my symptoms improve	242 (52.5)	120 (26.0)	99 (21.5)
P4	Before using antibiotics, I verify the expiration date	418 (90.7)	24 (5.2)	19 (4.1)
P5^a^	I prefer taking antibiotics when I experience a cough and sore throat	160 (34.7)	188 (40.8)	113 (24.5)

^a^Item has a reverse coding.

**Table 3:** Level of knowledge, attitude, and practice regarding antibiotic resistance.

**KAP attributes**	**Frequency (** **n** **)**	**Percentage (95% CI)**
*Knowledge*		
Lower	155	33.6 (29.6–38.3)
Moderate	208	45.1 (40.1–49.5)
High	98	21.3 (17.5–24.7)
*Attitude*		
Negative	214	46.4 (41.8–51.1)
Positive	247	53.6 (48.9–58.2)
*Practice*		
Poor practice	230	49.9 (45.6–54.2)
Good practice	231	50.1 (45.8–54.4)

**Table 4:** Factors associated with knowledge, attitude, and practice regarding antibiotics.

**Variable**	**Knowledge**			**χ** ^2^ ** (** **p** ** value)**	**Attitude**		**χ** ^2^ ** (** **p** ** value)**	**Practice**		**χ** ^2^ ** (** **p** ** value)**
	**High (%)**	**Moderate (%)**	**Low (%)**		**Positive (%)**	**Negative (%)**		**Good (%)**	**Poor (%)**	
*Age*										
< 20 years	10 (23.8)	17 (40.5)	15 (35.7)	1.21 (0.877)	21 (50.0)	21 (50.0)	0.37 (0.83)	29 (69.0)	13 (31.0)	7.45 (0.024)⁣^∗^
20–23 years	81 (21.4)	173 (45.8)	124 (32.8)		205 (54.2)	173 (45.8)		185 (48.9)	193 (51.1)	
> 23 years	7 (17.1)	18 (43.9)	16 (39.0)		21 (51.2)	20 (48.8)		17 (41.5)	24 (58.5)	
*Gender*										
Male	52 (24.2)	111 (51.6)	52 (24.2)	16.08 (< 0.001)⁣^∗∗∗^	113 (52.6)	102 (47.4)	0.17 (0.681)	100 (46.5)	115 (53.5)	2.08 (0.149)
Female	46 (18.7)	97 (39.4)	103 (33.6)		134 (54.5)	112 (45.5)		131 (53.3)	115 (46.7)	
*Academic discipline*										
Health sciences	37 (67.3)	18 (32.7)	0 (0.0)	88.80 (< 0.001)⁣^∗∗∗^	45 (81.8)	10 (18.2)	21.13 (< 0.001)⁣^∗∗∗^	34 (61.8)	21 (38.2)	6.54 (0.162)
Engineering	10 (11.9)	43 (51.2)	31 (36.9)		46 (54.8)	38 (45.2)		47 (56.0)	37 (44.0)	
Management	29 (14.0)	92 (44.4)	86 (41.5)		101 (48.8)	106 (51.2)		100 (48.3)	107 (51.7)	
Allied sciences	10 (17.5)	28 (49.1)	19 (33.3)		27 (47.4)	30 (52.6)		24 (42.1)	33 (57.9)	
Others	12 (20.7)	27 (46.6)	19 (32.8)		28 (48.3)	30 (51.7)		26 (44.8)	32 (55.2)	
*Studied antibiotics in the past*										
Yes	66 (27.7)	94 (39.5)	78 (32.8)	13.25 (0.001)⁣^∗∗^	141 (59.2)	97 (40.8)	6.37 (< 0.012)⁣^∗^	131 (55.0)	107 (45.0)	4.79 (0.029)⁣^∗^
No	32 (14.3)	114 (51.1)	77 (34.5)		106 (47.5)	117 (52.5)		100 (44.8)	123 (55.2)	

⁣^∗^Statistically significant at *p* < 0.05

⁣^∗∗^*p* < 0.01.

⁣^∗∗∗^*p* < 0.001.

**Table 5:** Relationship between antibiotic use practice with knowledge and attitude toward antibiotics.

**Variable**	**Knowledge**			**χ** ^2^ ** (** **p** ** value)**	**Attitude**		**χ** ^2^ ** (** **p** ** value)**
	**High (%)**	**Moderate (%)**	**Low (%)**		**Positive (%)**	**Negative (%)**	
*Consult a doctor for antibiotics*							
Always or usually	80 (23.1)	153 (44.2)	113 (32.7)	3.732 (0.443)	203 (58.7)	143 (41.3)	14.521 (0.001)⁣^∗∗^
Sometimes	15 (16.7)	44 (48.9)	31 (34.4)		35 (38.9)	55 (61.1)	
Seldom or never	3 (12.0)	11 (44.0)	11 (44.0)		9 (36.0)	16 (64.0)	
*Take proper counseling on antibiotics*							
Always or usually	73 (22.3)	138 (42.2)	116 (35.5)	4.516 (0.341)	179 (54.7)	148 (45.3)	0.657 (0.72)
Sometimes	14 (17.1)	45 (54.9)	23 (28.0)		41 (50.0)	41 (50.0)	
Seldom or never	11 (21.2)	25 (48.1)	16 (30.8)		27 (51.9)	25 (48.1)	
*Finish course of antibiotic treatment*							
Always or usually	66 (27.3)	108 (44.6)	68 (28.1)	14.758 (0.005)⁣^∗∗^	159 (65.7)	83 (34.3)	30.106 (< 0.001)⁣^∗∗∗^
Sometimes	14 (11.7)	56 (46.7)	50 (41.7)		48 (40.0)	72 (60.0)	
Seldom or never	18 (18.2)	44 (44.4)	37 (37.4)		40 (40.4)	59 (59.0)	
*Check the expiry dates of antibiotics*							
Always or usually	93 (22.2)	188 (45.0)	137 (32.8)	3.395^#^ (0.494)	228 (54.5)	190 (45.5)	1.742 (0.419)
Sometimes	3 (12.5)	11 (45.8)	10 (41.7)		11 (45.8)	13 (54.2)	
Seldom or never	2 (10.5)	9 (47.4)	8 (42.1)		8 (42.1)	11 (57.9)	
*Prefer antibiotics for cold*							
Always or usually	31 (19.4)	77 (48.1)	52 (32.5)	11.24 (0.024)⁣^∗^	78 (48.8)	82 (51.3)	3.48 (0.176)
Sometimes	37 (19.7)	74 (39.4)	77 (41.0)		101 (53.7)	87 (46.3)	
Seldom or never	30 (26.5)	57 (50.4)	26 (23.0)		68 (60.2)	45 (39.8)	

⁣^∗^Statistically significant at *p* < 0.05.

⁣^∗∗^*p* < 0.01.

⁣^∗∗∗^*p* < 0.001.

^#^Likelihood ratio.

## Data Availability

The data that support the findings of this study are available from the corresponding author upon reasonable request.
