# Supplementary material for: Knowledge, Attitude, and Practice on Antibiotic Use and Resistance Among Undergraduates, Pokhara Metropolitan, Nepal
Source: Biomed Res Int. 2025 Feb 10;2025:9928264. doi: 10.1155/bmri/9928264 (PMC11832259; doi:10.1155/bmri/9928264)
Supplement: Supporting Information — Additional supporting information can be found online in the Supporting Information section. Data collection tool. [file 9928264.f1.pdf]

Knowledge, Attitude, and Practice on Antibiotic Use and Resistance among Undergraduates, Pokhara Metropolitan, Nepal.

Section 1: Socio-demographic Information

1. What is your age (in completed years)? ……………..
2. What is your Gender?
    1. Male
    2. Female
    3. Other
3. What is your academic major?
    1. Health Sciences (Nursing & Public Health)
    2. Engineering
    3. Management
    4. Allied Sciences
    5. Others (Humanities, Law, Education)
4. Have you studied about antibiotics in the past academic courses?
    1. Yes
    2. No

Section 2: Knowledge About Antibiotic Use and Resistance
Instructions: For each statement below, please select the option that best reflects what you believe is correct. Choose one response for each item.

**Statements**		**Strongly Disagree (1)**	**Disagree (2)**	**Neutral (3)**	**Agree (4)**	**Strongly Agree (5)**
K1	Various antibiotics are required to treat various ailments					
K2	Antibiotics demonstrate effectiveness in combating bacterial infections.					
K3	Antibiotics can eliminate essential bacteria that typically reside on the skin and within the gastrointestinal tract.					
K4*	Antibiotics help the recovery from all coughs and colds					
K5*	Antibiotics are effective against all coughs and colds					
K6*	Antibiotics work against viral infections.					
K7	Overuse of antibiotics can grow bacterial resistance towards antibiotics.					
K8	Antibiotic resistance is a global concern					
K9	Humans could develop resistance to antibiotics.					

K10*	The use of antibiotics in animals has no effect in humans.					
**Section 3: Attitude towards Antibiotic Use and Resistance**<br>**Instructions**: For each statement below, please select the option that best reflects what you believe is correct. Choose one response for each item.						
**Statements**		**Strongly Disagree (1)**	**Disagree (2)**	**Neutral (3)**	**Agree (4)**	**Strongly Agree (5)**
A1*	Taking antibiotics during a cold is crucial to prevent the progression of a more severe illness.					
A2	It is recommended to complete the entire course of antibiotics treatment even if I start to feel better.					
A3*	Antibiotics aid in a faster recovery when experiencing a fever.					
A4	Every time I use an antibiotic, I contribute to the emergence of antibiotic resistance.					
A5*	The omission of one or two doses does not lead to the emergence of antibiotic resistance.					
A6*	Antibiotics are widely utilized because they are considered safe medications.					
**Section 4: Practice of Antibiotic Use**<br>**Instructions**: For each statement below, please select the option that best reflects what you believe is correct. Choose one response for each item.						
**Practice of antibiotics use**		**Always (1)**	**Usually (2)**	**Sometimes (3)**	**Seldom (4)**	**Never (5)**
P1	I consult a Physician/Pharmacist before starting an antibiotic.					
P2	Take proper counseling from pharmacists on how to use antibiotics before buying the medicine.					
P3	I consistently finish the entire course of antibiotics, even if my symptoms improve.					
P4	Before using antibiotics, I verify the expiration date.					
P5*	I prefer taking antibiotics when I experience a cough and sore throat.					
